# Minimal risk of contrast-induced kidney injury in a randomly selected cohort with mildly reduced GFR

**DOI:** 10.1007/s00330-020-07429-w

**Published:** 2020-11-06

**Authors:** Jeanette Carlqvist, Ulf Nyman, Gunnar Sterner, John Brandberg, Erika Fagman, Mikael Hellström

**Affiliations:** 1 Department of Radiology, Institute of Clinical Sciences, Sahlgrenska Academy, University of Gothenburg, Gothenburg, Sweden; 2 Department of Radiology, Sahlgrenska University Hospital, Bruna Stråket 11B, 41345 Gothenburg, Sweden; 3 Department of Translational Medicine, Division of Medical Radiology, Skåne University Hospital, Lund University, Malmö, Sweden; 4 Department of Nephrology, Skåne University Hospital, Malmö, Sweden

**Keywords:** Acute kidney injury, Iohexol, Computed tomography angiography, Drug-related side effects and adverse reactions, Prospective studies

## Abstract

**Objectives:**

Previous large studies of contrast-induced or post-contrast acute kidney injury (CI-AKI/PC-AKI) have been observational, and mostly retrospective, often with patients undergoing non-enhanced CT as controls. This carries risk of inclusion bias that makes the true incidence of PC-AKI hard to interpret. Our aim was to determine the incidence of PC-AKI in a large, randomly selected cohort, comparing the serum creatinine (Scr) changes after contrast medium exposure with the normal intraindividual fluctuation in Scr.

**Methods:**

In this prospective study of 1009 participants (age 50–65 years, 48% females) in the Swedish CArdioPulmonary bioImage Study (SCAPIS), with estimated glomerular filtration rate (eGFR) ≥ 50 mL/min, all received standard dose intravenous iohexol at coronary CT angiography (CCTA). Two separate pre-CCTA Scr samples and a follow-up sample 2–4 days post-CCTA were obtained. Change in Scr was statistically analyzed and stratification was used in the search of possible risk factors.

**Results:**

Median increase of Scr post-CCTA was 0–2 μmol/L. PC-AKI was observed in 12/1009 individuals (1.2%) according to the old ESUR criteria (> 25% or > 44 μmol/L Scr increase) and 2 individuals (0.2%) when using the updated ESUR criteria (≥ 50% or ≥ 27 μmol/L Scr increase). Possible risk factors (e.g., diabetes, age, eGFR, NSAID use) did not show increased risk of developing PC-AKI. The mean effect of contrast media on Scr did not exceed the intraindividual Scr fluctuation.

**Conclusions:**

Iohexol administration to a randomly selected cohort with mildly reduced eGFR is safe, and PC-AKI is very rare, occurring in only 0.2% when applying the updated ESUR criteria.

**Key Points:**

*• Iohexol administration to a randomly selected cohort, 50–65 years old with mildly reduced eGFR, is safe and PC-AKI is very rare.*

*• Applying the updated ESUR PC-AKI criteria resulted in fewer cases, 0.2% compared to 1.2% using the old ESUR criteria in this cohort with predominantly mild reduction of renal function.*

*• The mean effect of CM on Scr did not exceed the intraindividual background fluctuation of Scr, regardless of potential risk factors, such as diabetes or NSAID use in our cohort of 1009 individuals.*

## Introduction

The real frequency and clinical importance of contrast induced acute kidney injury (CI-AKI) have been debated over the decades, with incidence data ranging from < 2 to 70% [1, 2]. Recent controlled studies indicate that the risk of CI-AKI has been overestimated [3]. Confounding issues by variations in indication for computed tomography (CT) and possible selection bias of control groups in these retrospective studies make it difficult to observe true effects of contrast media (CM) on renal function [4–7]. Moreover, AKI following CM examinations may be caused by other conditions temporally related to the administration of intravascular CM. Therefore, the term post-contrast AKI (PC-AKI) has been introduced for AKI regardless if CM was the cause [8]. Nevertheless, individual patient risk of CI-AKI is a daily concern for radiologists and referring clinicians.

Most patients with sufficient serum creatinine (Scr) data after CT examinations are inpatients, who are more likely to receive post-scan Scr monitoring as part of their medical care, compared to outpatients. These results may not be applicable to patients receiving intravenous contrast media (IV-CM) in an outpatient setting with normal to moderately reduced renal function [3, 9–11]. Studies of PC-AKI in mainly inpatients with estimated glomerular filtration rate (eGFR) > 60 mL/min/1.73 m^2^ showed incidences of 2.1% and 5.4% [12, 13]. Davenport et al [13] showed an AKI incidence of 10.5% among inpatients with eGFR 45–59 mL/min/1.73 m^2^, but no significant difference compared to a control group undergoing non-enhanced CT.

Varying definitions of CI-AKI, Scr measurement errors, and intraindividual variations in Scr levels add to the difficulties in understanding the effects of CM on renal function. Recently, the Contrast Media Safety Committee of European Society of Urogenital Radiology (ESUR) changed the Scr criteria for AKI by adopting those of KDIGO (Kidney Disease: Improving Global Outcomes) [14]. This may also lead to different frequency figures of post-contrast PC-AKI and CI-AKI, as compared to previous estimations.

In a large prospective multicenter screening study on biomarkers for cardiovascular and pulmonary disease (SCAPIS), coronary CT angiography (CCTA) was an integrated part [15]. Considering the uncertainty regarding PC-AKI/CI-AKI, it was therefore of interest to study possible effects of CM on renal function, by means of Scr measurement, in this screening study of individuals with mostly mildly reduced renal function. The aim was also to try to determine if any PC-AKI could be attributed to the CM administration (CI-AKI), applying the updated and older definitions of CI-AKI, by taking spontaneous variations in Scr into consideration.

## Materials and methods

### Participants

Participants were enrolled from the Swedish CArdioPulmonary bioImage Study (SCAPIS) including 30,000 participants aged 50–65 years. SCAPIS participants were randomly selected from the population registry and underwent extensive cardiovascular and pulmonary investigation, including CCTA [15]. For this study, participants from the Gothenburg cohort (total 6000 individuals) were eligible for inclusion from October 2014 to September 2016. The study was approved by the National Research Ethics Committee in Umeå (2014-02-14: reference number 2014-33-32M) and the Regional Research Ethics Committee in Gothenburg (2018-10-19: reference number 848-18) (for background data from SCAPIS data bank). All participants provided signed informed consent.

As per the SCAPIS protocol, only individuals with eGFR ≥ 50 mL/min, estimated by the absolute Lund-Malmö creatinine equation [16, 17], were eligible for CCTA, and, thereby, for this study. This was based on the risk threshold for CI-AKI (GFR < 45 mL/min) recommended by the Swedish Society of Radiology [18]. Individuals who declined CM injection, had contraindications due to known CM hypersensitivity and severe allergy to other substances, or stated that they would not be willing or able to provide a follow-up blood sample 48–96 h after the CCTA examination were excluded. Enrollment continued until a study sample of 1000 evaluable participants was reached, as calculated by pre-study power estimation for CI-AKI (> 44 μmol/L or > 25% Scr increase compared to baseline).

European Society of Urogenital Radiology (ESUR) PC-AKI criteria:

Updated PC-AKI criteria: Scr increase of ≥ 27 μmol/L or ≥ 50% compared to baseline [8].

Old PC-AKI criteria: Scr increase of > 44 μmol/L or > 25% compared to baseline [19].

For practical reasons, we extended the post-CCTA Scr blood sampling period to 48–96 h, while the old and updated ESUR criteria stipulate 48–72 h.

### Endpoints

The primary endpoint was the frequency of PC-AKI, based on the old and updated ESUR criteria, respectively.

Secondary endpoints were the mean change in Scr between the two blood samples pre-CCTA, i.e., the intraindividual variation in Scr; the mean change in Scr between the pre-CCTA samples and the post-CCTA sample; the frequency of CI-AKI among the PC-AKI cases, after alternative causes for PC-AKI had been excluded by telephone interviews and search for comorbidities in the medical SCAPIS files; and the mean change in Scr related to the number of AKI risk factors.

### Pre-CCTA serum creatinine sampling

The first pre-CCTA Scr sampling was obtained as part of the SCAPIS protocol and was used for calculation of eGFR. Study participants were instructed not to eat for 10 h prior to the blood sampling performed in the morning but were allowed to drink freely. This blood sampling was performed at a median of 14 days (range 0–91 days; 2.5th and 97.5th percentiles: 3 days and 70 days, respectively) before the CCTA.

A second pre-CCTA Scr sampling was obtained immediately prior to CCTA. The examinations were performed between 8 am and 3 pm and the participants were instructed not to eat for 4 h before the CCTA but to drink 300 mL of a nutritional beverage, for other research purposes within SCAPIS, 1 h prior to the examination and at least 2 dL water as pre-CCTA hydration.

### CCTA examination

All participants received 325 mg I/kg of iohexol (Omnipaque 350 mg/mL, GE Healthcare AB), with a minimum/maximum dosing weight of 50/80 kg for women and 50/90 kg for men followed by a flush injection of 60 mL 0.9% NaCl. Contrast injection time was set to 12 s. A 10 mL contrast medium test bolus followed by a similar NaCl flush injection was used to optimize scan timing in the arterial phase. The mean CM dose was 29 (range 20–43) grams of iodine and the mean gram-iodine/eGFR ratio was 0.35 (range 0.22–0.59).

### Post-CCTA Scr sampling

Samples were obtained 48–96 h after CCTA at any time during the day without food or liquid restrictions, except to avoid meals rich in meat 8 hours before Scr sampling in order to avoid Scr increase due to heavy meat intake [20–23].

Individuals developing PC-AKI were contacted for additional Scr follow-ups until return to baseline Scr values. A telephone interview, in addition to checking their medical files, was performed regarding potential clinical symptoms or factors other than CM exposure that could explain the Scr increase. In addition, the common data bank of the included chemical laboratories was scrutinized 1.5 years after last participant inclusion to identify any potential cases of renal failure within the study cohort (*n* = 1551).

### Serum creatinine analysis

All blood samples were analyzed with an enzymatic assay on a Cobas 8000 (CREA Creatinine plus, Roche Diagnostics) at one of three accredited clinical chemistry laboratories at the hospital. The method is traceable to primary reference material with values assigned by isotope dilution mass spectrometry (IDMS, National Institute of Standards and Technology, SRM 967). The total coefficient of variation (CV) for the creatinine method was 1.2% at 92 μmol/L and 0.9% at 346 μmol/L.

### Background risk factors and generalizability

Data about potential risk factors, apart from renal function, for developing PC-AKI, i.e., age (below or above 57 years), diabetes mellitus, cardiovascular disease (measured as coronary calcification score, CACS, or hypertension defined as systolic blood pressure > 140 or diastolic > 70 mmHg, or confirmed history of hypertension), current medication with non-steroidal anti-inflammatory drugs (NSAID), and CM dose (including gram-iodine/eGFR ratio) [24], were provided from the SCAPIS databank. To study the representativeness of our study cohort, these background data were compared with background data from the remaining 5255 participants of the Gothenburg SCAPIS cohort.

### Statistical methods

For the analysis of change in Scr, we used a mixed model with repeated measurements, where confidence intervals and *p* values were adjusted for multiple comparisons using the Tukey-Kramer method. For comparisons between groups, the chi-square test or Fisher’s exact test was used for proportions and Students *t* test or Mann-Whitney *U* test for continuous/ordered variables. All tests are two-sided and *p* values below 0.05 were considered statistically significant. Analyses were performed using SAS for Windows version 9.4.

#### Study cohort characteristics

**Fig. 1:**
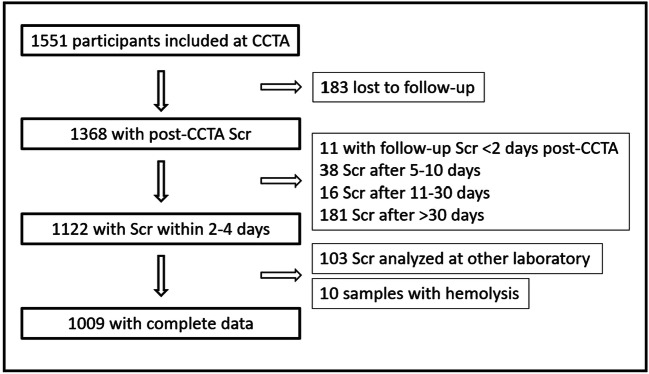
Flow chart of study population. CCTA, coronary CT angiography

There were 1551 SCAPIS participants initially included with two separate Scr samples pre-CCTA. Follow-up Scr was available for 1368 individuals (88%). As shown in Fig. 1, the final analysis with post-CCTA Scr samples taken per protocol included 1009 individuals, 528 males and 481 females. Individual characteristics are presented in Table 1. The majority of the participants (89.3%) had mildly reduced renal function (eGFR 60–89 mL/min/1.73 m^2^), 5.4% had normal function (eGFR ≥ 90 mL/min/1.73 m^2^), and 5.3% had mildly to moderately reduced function (eGFR 45–59 mL/min/1.73 m^2^) according to the KDIGO criteria [25]. When comparing our study cohort (*n* = 1009) with the entire SCAPIS Gothenburg cohort (*n* = 5255), the cohorts were in most aspects comparable, with few significant differences (Table 1). There was a slightly higher proportion of men in our study cohort than in the entire SCAPIS cohort (52.3% compared to 46.8%, *p* = 0.001, std. diff 0.11).

**Table 1:** Baseline comparison of the study cohort and the remaining Gothenburg SCAPIS cohort

	Study cohort	Remaining Gothenburg SCAPIS cohort	*p* value	Standardized difference (absolute)
Total no. of individuals	1009	5255		
Female sex	481 (47.7)	2795 (53.2)	0.001	0.11
Age, year	57.7/57.8/50.1–65.2	57.4/57.3/50.1–65.5	0.05	0.07
CACS [23/234]	68.7/0/0–3587	56.8/0/0–5213	0.14	0.05
CACS > 0	396 (40.2)	2011 (40.1)	0.97	0.00
Hypertension^‡^ [27/117]	278 (28.3)	1566 (30.5)	0.18	0.05
Smokers/ex-smokers [22/123]	522 (52.9)	2740 (53.4)	0.78	0.01
Diabetes [2/58]	79 (7.8)	335 (6.4)	0.11	0.05
NSAID past 2 weeks [36/183]	302 (31.0)	1504 (29.7)	0.40	0.03
Body mass index, kg/m^2^	26.8/26.2/16.4–46.1	26.8/26.2/15.4–50.6	0.92	0.00
Serum creatinine (μmol/L) [0/25]	79.1/79/42–138	78.4/77/34–340	0.19	0.05
eGFR, mL/min [0/25]	83.2/82/52–148	82.5/81/17–178	0.32	0.03

*p* values less than 0.05 indicate a statistically significant difference. Continuous variables reported as mean/median/range. Numbers in parentheses are percentages. Number of individuals with missing data from study cohort/remaining SCAPIS indicated by brackets

*CACS*, coronary artery calcification score

^‡^Hypertension defined as systolic blood pressure > 140 or diastolic > 70 mmHg or confirmed history of hypertension

## Results

**Fig. 2:**
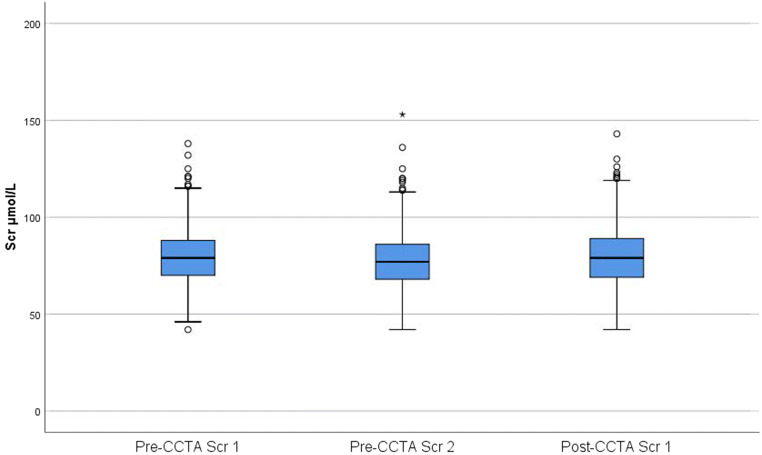
Scr levels for the entire study population (*n* = 1009) at the three separate Scr samplings, two samples pre- and one sample post-coronary CT angiography (CCTA). Box plots demonstrating Scr levels, 50% of Scr values within boxes (median value plotted as horizontal line); whiskers represent 95% confidence interval and outliers are indicated by circles and extreme outlier (> 1.5 × interquartile range) (*n* = 1) by asterisk

For the whole cohort, there was a significant, yet small, variation in mean Scr between the three different samplings (Fig. 2; Table 2). Comparing the two pre-CCTA samples for intraindividual Scr fluctuations prior to CM injection, none of the participants showed an increase in Scr reaching a level corresponding to either of the two definitions of PC-AKI.

**Table 2:** Serum creatinine (Scr) change observed between the three Scr sampling occasions

Scr (*n* = 1009)	Mean difference (μmol/L)	Standard error	Adjusted *p* value	Adjusted 95% CI	Median difference (μmol/L)	Percentile 2.5th/97.5th
Scr 2 – Scr 1	− 1.21	0.18	< 0.0001	− 1.64, − 0.79	− 1	− 13.0/10.0
Scr 3 – Scr 1	0.70	0.22	0.004	0.19, 1.20	0	− 12.0/15.0
Scr 3 – Scr 2	1.91	0.20	< 0.0001	1.43, 2.39	2	− 10.0/15.8

A statistically significant, but small and clinically insignificant, difference between pre- and post-CCTA Scr values was shown

Scr 1: First Scr sample pre-CCTA (median 14 days before CCTA)

Scr 2: Second Scr sample pre-CCTA (same day as CCTA)

Scr 3: Scr 48–96 h post-CCTA

*CCTA*, coronary computed tomography angiography

Using the Scr values taken immediately prior to CCTA as baseline (second pre-CCTA sample), PC-AKI by the updated criteria (Scr increase of ≥ 27 μmol/L or ≥ 50%) was observed in two of 1009 participants (0.2%). The old criteria for PC-AKI (> 44 μmol/L or > 25% increase in Scr) rendered 12 cases (1.2%). When using the first pre-CCTA Scr value as baseline, 10 participants (1.0%) had PC-AKI according to the older criteria and among these four individuals (0.4%) fulfilled the updated PC-AKI criteria (Table 3). The Scr values returned to baseline in all but one individual at follow-up sampling (Table 4). This individual, with an isolated low Scr at second pre-CCTA blood sampling (case 5), showed persistent Scr levels at second follow-up sample, however stable compared to the first pre-CCTA Scr value. One individual could not be reached for follow-up. The 12 PC-AKI (old ESUR criteria) cases did not show any significant difference compared to the remaining study cohort, in terms of potential risk factors; presence of diabetes, cardiovascular disease (measured as CACS), and hypertension; use of NSAID; or eGFR. Ten of the 12 participants could be reached for telephone interviews, which revealed one possible case of dehydration at the time of follow-up Scr sample (case no. 2). No disorders or circumstances other than CM as the cause of PC-AKI were found in the remaining nine cases. Consequently, these were considered as CI-AKI cases.

**Table 3:** Number of post-contrast acute kidney injury (PC-AKI) cases based on the updated and the old criteria by the European Society of Urogenital Radiology (ESUR), relative to the first and second serum creatinine (Scr) measurements prior to coronary CT angiography (CCTA)

	Definition of PC-AKI	Pre-CCTA Scr1 as baseline (number of cases)	Pre-CCTA Scr2 as baseline (number of cases)
Updated ESUR PC-AKI criteria	≥ 27 μmol/L Scr increase from baseline	4	2
	≥ 50% Scr increase from baseline	0	1*
Old ESUR PC-AKI criteria	> 44 μmol/L Scr increase from baseline	0	0
	> 25% Scr increase from baseline	10	12

*This individual fulfilled both relative and absolute Scr criteria according to the updated ESUR PC-AKI definition

**Table 4:** Pre- and post-coronary CT angiography (CCTA) Scr values in the 12 individuals who developed PC-AKI according to the old ESUR PC-AKI criteria

		Pre-CCTAScr 1	Pre-CCTAScr 2	Post-CCTAScr 3	Post-CCTAScr 4	Post-CCTAScr 5	Post-studyScr 6	Post-studyScr 7
1	Scr (μmol/L)	56	57	73	63			
	Days	− 23	0	2	6			
2	Scr (μmol/L)	71	78	104	100			
	Days	− 13	0	4	11			
3	Scr (μmol/L)	84	76	96			89	90
	Days	− 15	0	4			887	902
4	Scr (μmol/L)	74	59	81	62	76	67	
	Days	− 1	0	3	17	20	93	
5	Scr (μmol/L)	104	60	102*	105	103		
	Days	− 28	0	3	12	39		
6	Scr (μmol/L)	90	92	130*	98		107	98
	Days	− 10	0	4	13		203	236
7	Scr (μmol/L)	67	52	76				
	Days	− 21	0	2				
8	Scr (μmol/L)	79	64	87	76			
	Days	− 11	0	3	22			
9	Scr (μmol/L)	65	64	82	66			
	Days	− 11	0	2	7			
10	Scr (μmol/L)	67	67	89	72		51	
	Days	− 28	0	2	31		1075	
11	Scr (μmol/L)	72	72	91	74			
	Days	− 42	0	4	12			
12	Scr (μmol/L)	73	68	88	73		67	
	Days	− 21	0	2	12		259	

Days: number of days prior to and after CCTA

*Individuals fulfilling criteria for the updated PC-AKI definition (≥ 50% or ≥ 27 μmol/L Scr increase)

An additional control of Scr in Sahlgrenska University Hospital’s laboratory data bank, for the whole cohort of 1551 participants 1.5 years after last inclusion, showed no case of renal injury or failure (Scr increase 2 or 3 times, respectively) according to the RIFLE criteria [26].

**Fig. 3:**
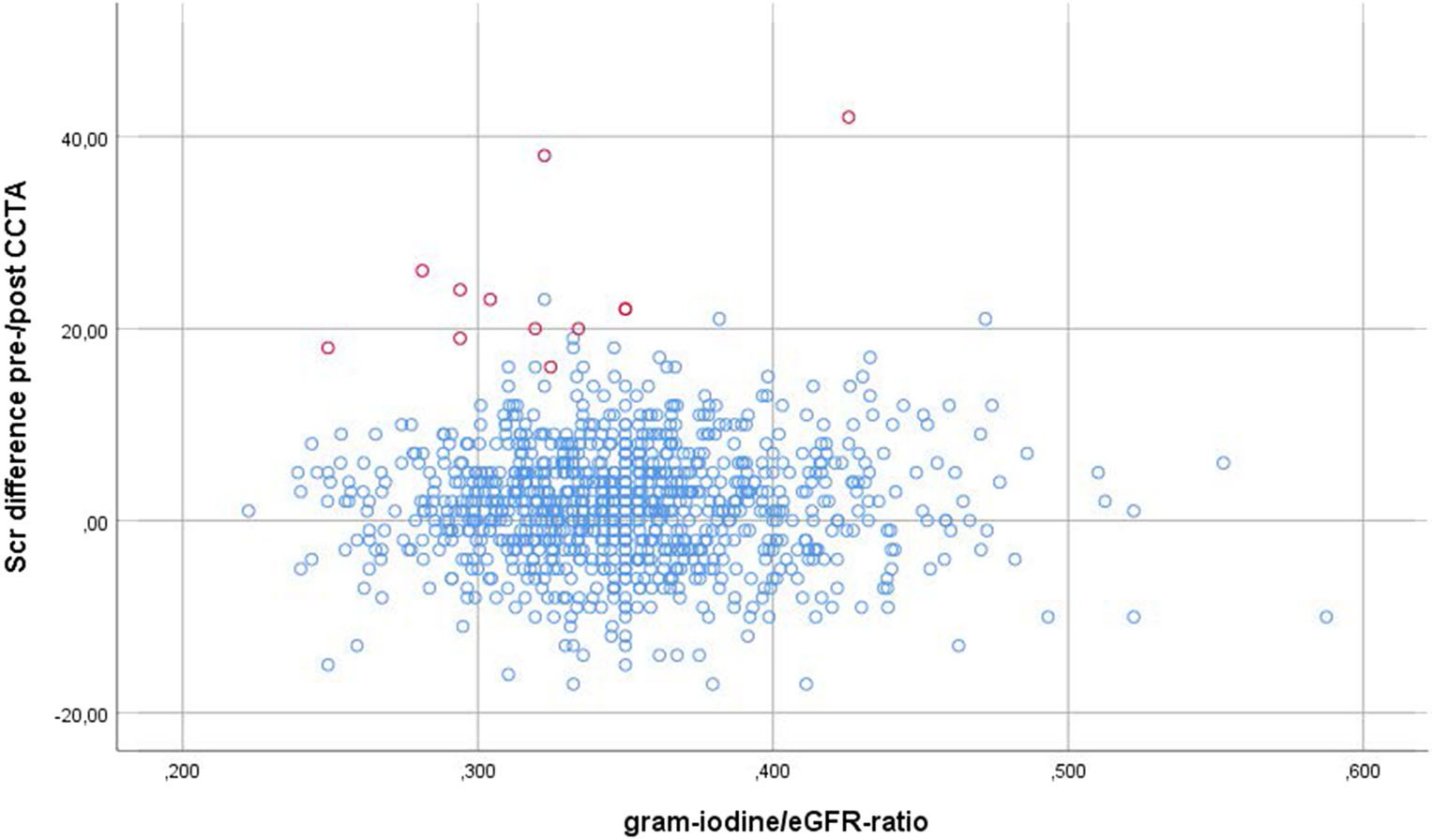
Serum creatinine (Scr) difference pre- compared to post-CCTA (coronary CT angiography) in relation to gram-iodine/eGFR (estimated glomerular filtration rate) ratio. Post-contrast acute kidney injury (PC-AKI) cases (> 25% Scr increase) indicated in red

When stratifying on possible risk factors such as age (above/below 57 years), renal function (eGFR above/below 70 mL/min), diabetes, cardiovascular risk factors (CACS, hypertension), use of NSAID past 2 weeks, high vs low volume of administered CM (≤ 29 vs > 29 g iodine), and gram-iodine/eGFR ratio (plotted in Fig. 3), no correlation or statistical significance was observed for any of these variables in terms of Scr increase. There was no trend of multiple risk factors aggravating Scr increase, as shown in Table 5.

**Table 5:** Serum creatinine (Scr) changes post-CCTA (coronary CT angiography) compared with 2nd pre-CCTA Scr values, stratified by number of risk factors

No. of risk factors	Number^#^ (%)	Serum creatinine change (μmol/L)		*p* value^##^
		Mean ± SD	Median (10th, 90th percentiles)	
Absolute Scr change				
0 risk factor	296 (30.5)	1.9 ± 7.2	2 (− 6, 10)	
1 risk factor	476 (49.0)	1.7 ± 6.2	2 (− 6, 9)	0.98
2 risk factors	183 (18.8)	2.1 ± 5.8	2 (− 5, 10)	0.59
3–4** risk factors	17 (1.7)	2.8 ± 7.5	5 (− 10, 11)	0.28
Relative Scr change (%)				
0 risk factor	296 (30.5)	2.9 ± 9.4	2 (− 7, 13)	
1 risk factor	476 (49.0)	2.6 ± 8.3	2 (− 7, 13)	0.98
2 risk factors	183 (18.8)	2.9 ± 7.6	3 (− 6, 13)	0.63
3–4** risk factors	17 (1.7)	4.5 ± 9.2	6 (− 9, 17)	0.27

Risk factors: age > 57 years, NSAID past 2 weeks, eGFR < 60 mL/min, and diabetes

**Three and four risk factors combined since only one individual had all four risk factors

^#^Thirty-seven individuals with missing data on NSAID and/or diabetes excluded

^##^Compared to group with zero risk factors

## Discussion

This prospective study of 1009 randomly selected individuals aged 50–65 years within the SCAPIS study, the vast majority (89%) with mildly reduced renal function, showed a statistically significant but minimal mean increase in Scr 48–96 h after CCTA with intravenous CM exposure. This suggests a real, but transient, and clinically insignificant effect of CM on renal function. In fact, the mean Scr increase after CM administration was only 1.9 μmol/L, i.e., of the same magnitude as the intraindividual difference between the two pre-CCTA Scr samples (mean 1.2 μmol/L). This magnitude of Scr variation can also be considered to be within normal intraindividual diurnal and day-to-day variation [27, 28].

Using the old or updated ESUR criteria for PC-AKI, the incidence was low, 1.2% and 0.2% respectively. Previous studies have shown similar or higher PC-AKI incidence for individuals with mildly reduced renal function [12, 24, 29]. However, most previous studies were hampered by their retrospective design using control groups consisting of patients undergoing CT without CM, although propensity scoring or other measures were taken to reduce selection bias. We were able to assess the effects of CM on renal function before and after intravenous CM administration without the confounding issues of selection bias, such as differences in comorbidity at inclusion. As a substitute for a control group, we used repeated individual measurements of Scr prior to CM injection, ensuring stable Scr levels before CM administration. The Scr changes post-CCTA could thus be compared to the intraindividual baseline Scr level fluctuations, eliminating background fluctuations of Scr as a potential source of error [30].

In a clinical context, it is often difficult to determine if an increase in Scr after CM administration is due to the CM itself, or to any other condition temporally related to the administration of CM. In retrospective controlled studies, selection bias is an issue and their evidence value has been graded as low [31]. In one propensity matched controlled study, the etiology of AKI following chart review was hemodynamic in 80% of the cases in the non-enhanced CT group but only 8% in the CM-enhanced CT group with CM being the only likely etiological factor in 52% of the PC-AKI cases [32]. In our study, the participants were their own controls and in no case did the pre-CCTA Scr change reach a magnitude corresponding to the PC-AKI criteria. In addition, among the 12 cases with PC-AKI, we could not find any alternative etiological factor based on chart review and telephone interviews, except for one case with possible dehydration at post-CM Scr sampling (case 2). We therefore conclude that the frequency figures of PC-AKI (0.2% by the updated criteria or 1.2% by the old ESUR criteria) most likely represent CI-AKI in this cohort. Importantly, the rise in Scr was temporary, and in only three cases did it reach above the normal reference values for Scr. Only one individual had a persistent elevation of Scr at a second follow-up sampling, but it was stable if using the first pre-CCTA sample as baseline, likely indicating a measurement error of the second pre-CCTA sample. These results are important for risk estimation of outpatients with mildly reduced renal function who undergo CM examinations at a gram-iodine/GFR ratio below 0.6, and comforting in a screening scenario where intravenous CM is used. Thereby, it also functions as a safety assessment of CCTA in the SCAPIS study, where a total of 30,000 individuals were exposed to CM.

Applying the updated ESUR PC-AKI criteria seems to lead to fewer cases of PC-AKI as compared to the old criteria (only two individuals compared to twelve). It is noteworthy that, using the old criteria, all 12 cases fulfilled the relative criterion (> 25% increase) and none fulfilled the absolute criterion (> 44 μmol/L increase), while using the updated criteria the two cases fulfilled the absolute criterion (≥ 27 μmol/L increase; Table 3) and one of them also fulfilled the relative criterion (≥ 50% increase). Ninety-eight percent of the baseline Scr values in the present study were below 108 μmol/L; the upper threshold for a 25% increase from 108 μmol/L equals 27 μmol/L. Thus, in the vast majority of the participants, a 25% increase was a more sensitive criterion to diagnose AKI than 27 μmol/L.

We are not aware of any studies that provide conclusive evidence about risk factors for development of PC-AKI in patients with only mildly reduced renal function. With the available comprehensive information of the study participants’ medical history and standardized tests for, e.g., diabetes, blood pressure, CACS, and body mass index supplied by SCAPIS, we were able to analyze the impact of associated known or presumed risk factors for PC-AKI. None of the analyzed risk factors could be associated with an increase of Scr after CM administration. This may be explained by the small effects on Scr in this study and does not rule out an impact of such risk factors in patients with more severely reduced renal function and more advanced risk factors [18]. In a meta-analysis focused on risk factors for contrast-induced nephropathy, Moos et al [33] concluded that the risk factors “probably related” to contrast-induced nephropathy are pre-existing renal insufficiency, old age, diabetes, and use of nephrotoxic drugs. However, their meta-analysis was not stratified according to GFR levels, and therefore, no conclusions regarding those with only mild renal insufficiency can be drawn.

Our study cohort consisted mostly of individuals with mildly reduced renal function, and it did not differ from the entire SCAPIS cohort in terms of eGFR levels or potential PC-AKI risk factors (Table 1). The fact that diabetes, hypertension, and NSAID use was fairly frequent in our cohort, 7.8%, 28.3%, and 31.0%, respectively, implies that it could be representative for the age span 50–65 years in the general population or an outpatient clinical setting, although one could argue that patients seeking medical care may have additional risk factors for PC-AKI. Furthermore, with a mean iodine dose of 29 g and mean gram-iodine/eGFR ratio of 0.35, the CM doses within this study can be considered as moderate [18]. The results may therefore not be applicable to patients receiving higher CM doses.

Our study was limited to those with eGFR ≥ 50 mL/min. It should also be acknowledged that estimations of GFR based on Scr are prone to large variations, as compared to measured GFR by, e.g., iohexol clearance. Thus, 15–25% of GFR estimations are outside ± 30% of measured GFR values [17]. We used the absolute Lund-Malmö eGFR equation, which has been shown to outperform both MDRD (Modification of Diet in Renal Disease study) and CKD-EPI (Chronic Kidney Disease Epidemiology collaboration) in a Swedish cohort, especially in the GFR interval 60–89 mL/min, using plasma clearance of iohexol as reference [17]. Another limitation of our study is that we only studied individuals in the age range 50–65 years; i.e., no young adults or elderly were included. Thus, our results are not necessarily generalizable to these age categories.

We had missing follow-up Scr samples for 12% of our intended study population. The additional control of the entire cohort (*n* = 1551) in the hospital’s laboratory data bank 1.5 years after last inclusion showed no cases of severe renal failure. Since all study participants resided in Gothenburg, they would reasonably have been admitted to these laboratories in case of severe renal failure.

In conclusion, the risk of PC-AKI in this randomly selected cohort was very low (1.2%), even lower when using the updated ESUR criteria (0.2%). Our findings are consistent with previous studies showing little risk in patients with eGFR ≥ 45 mL/min/1.73 m^2^ [12, 24, 29]. Individuals with potential risk factors, such as diabetes or NSAID use, did not show more pronounced Scr increase after CM exposure compared to those without risk factors. The mean effect of CM on Scr did not exceed the intraindividual Scr fluctuation. Intravenous iohexol administration seems safe for individuals with mildly reduced renal function with the present CM dose and a gram-iodine dose/eGFR ratio below 0.6. Applying the updated PC-AKI criteria by ESUR results in fewer cases of PC-AKI, as compared to the old criteria.

## Abbreviations

CACS: Coronary artery calcium scoring
CCTA: Coronary computed tomography angiography
CI-AKI: Contrast-induced acute kidney injury
CM: Contrast media
eGFR: Estimated glomerular filtration rate
ESUR: European Society of Urogenital Radiology
KDIGO: Kidney Disease: Improving Global Outcomes
PC-AKI: Post-contrast acute kidney injury
RIFLE: Risk of renal dysfunction, Injury to the kidney, Failure of kidney function, Loss of kidney function, and End-stage kidney disease
SCAPIS: Swedish CArdioPulmonary bioImage Study
Scr: Serum creatinine
